# *De Novo* Assembly of Coding Sequences of the Mangrove Palm (*Nypa fruticans*) Using RNA-Seq and Discovery of Whole-Genome Duplications in the Ancestor of Palms

**DOI:** 10.1371/journal.pone.0145385

**Published:** 2015-12-18

**Authors:** Ziwen He, Zhang Zhang, Wuxia Guo, Ying Zhang, Renchao Zhou, Suhua Shi

**Affiliations:** 1 State Key Laboratory of Biocontrol, Guangdong Provincial Key Laboratory of Plant Resources, Key Laboratory of Biodiversity Dynamics and Conservation of Guangdong Higher Education Institutes, Sun Yat-Sen University, Guangzhou, Guangdong 510275, China; 2 College of Life Sciences, Hainan Normal University, Haikou, Hainan 571158, China; University of Western Sydney, AUSTRALIA

## Abstract

*Nypa fruticans* (Arecaceae) is the only monocot species of true mangroves. This species represents the earliest mangrove fossil recorded. How *N*. *fruticans* adapts to the harsh and unstable intertidal zone is an interesting question. However, the 60 gene segments deposited in NCBI are insufficient for solving this question. In this study, we sequenced, assembled and annotated the transcriptome of *N*. *fruticans* using next-generation sequencing technology. A total of 19,918,800 clean paired-end reads were *de novo* assembled into 45,368 unigenes with a N50 length of 1,096 bp. A total of 41.35% unigenes were functionally annotated using Blast2GO. Many genes annotated to “response to stress” and 15 putative positively selected genes were identified. Simple sequence repeats were identified and compared with other palms. The divergence time between *N*. *fruticans* and other palms was estimated at 75 million years ago using the genomic data, which is consistent with the fossil record. After calculating the synonymous substitution rate between paralogs, we found that two whole-genome duplication events were shared by *N*. *fruticans* and other palms. These duplication events provided a large amount of raw material for the more than 2,000 later speciation events in Arecaceae. This study provides a high quality resource for further functional and evolutionary studies of *N*. *fruticans* and palms in general.

## Introduction

Mangroves belong to different families or orders but have adapted to the same unstable environment of intertidal zones [1]. Among the approximately 70 mangrove species, the “mangrove palm” *Nypa fruticans* (Thunb.) Wurmb. is the only monocot species [1, 2]. This species is the sole species of genus *Nypa* and a basal species in the family Arecaceae (exclude subfamily Calamoideae) [3]. *N*. *fruticans* is naturally distributed in the Old World from Ceylon, the Ganges Delta, Burma, and the Malay Peninsula through Indonesia to New Guinea and the Solomon Islands, northward to the Philippines and Ryukyu Islands, and southward to northern Queensland [1]. *N*. *fruticans* was recently reported as an invasive species in West Africa [4]. However, its fossils had a nearly cosmopolitan distribution throughout the Eocene [2]. The earliest pollen fossil of *Nypa* was found in present-day Egypt [5] and was dated to the Campanian (Late Cretaceous; approximately 75 million years ago), which is the oldest mangrove species fossil. The first fruit fossil of *Nypa* was found in the Paleocene [6]. Throughout its long-term evolutionary history, *N*. *fruticans* has developed many adaptive traits (such as vivipary and floating fruits) to survive in high-saline and hypoxic habitats. Vivipary is a special characteristic. The embryo that results from normal sexual reproduction has no dormancy but grows out of fruit coat while still attached to the parent plant [1], which is important for seedlings to avoid high saline environment. The mature fruits of *N*. *fruticans* are dispersed by floating on the water and movements caused by the tides aids the detachment of mature fruits. *N*. *fruticans* differs from most other palms because it lacks an erect stem. Instead, its thick, dichotomously branched, rhizomatous axis grows buried in mud and exhibits a special colonial growth habit that forms pure stands in brackish waters such as estuaries and lagoons [2]. Despite its evolutionary significance, few studies have been conducted on its characteristic. A recent study showed an extremely low diversity within and among six populations in China, Vietnam and Thailand using SSR (simple sequence repeat) and ISSR (inter-simple sequence repeat) analysis and suggested that bottlenecks in glacial epochs, founder effects and the mode of propagation have resulted in its low diversity [7].

Why *N*. *fruticans* is the only monocot species that could adapt to unstable intertidal zones represents an interesting question. Natural selection under different ecological environments would have driven the evolution of *N*. *fruticans* and its divergence from terraneous palms. Due to the rapid development of next-generation sequencing (NGS) technology, it is substantially easier to obtain resources, including genome data and transcriptome data, to explore unanswered evolutionary questions. In Arecaceae, the genomes of two species (date palm *Phoenix dactylifera* and oil palm *Elaeis guineensis*) and the transcriptome of *Cocos nucifera* have been sequenced [8–10]. However, only 60 nucleotide sequences of *N*. *fruticans* have been deposited in the NCBI database. This amount is insufficient for solving the questions of when *N*. *fruticans* diverged from its palm relatives and how this special and sole monocot adapts to mangrove habitats.

Whole-genome duplication (WGD), or polyploidy, has been recognized as an important event for plants. Nearly 15% of angiosperms and 31% of fern speciation events are related to ploidy increases [11]. Many duplication events have been detected in plants via synteny blocks of the whole genome [12] or paralogs identified via transcriptomes and ESTs [13, 14], which can provide many raw materials for their adaptation. Whether *N*. *fruticans* had a WGD can be inferred by genomic or transcriptomic datasets. Here, we present high quality *de novo* assembled and well-annotated transcriptome data for *N*. *fruticans* to study its adaptation to the intertidal zones, and shed light on the researches of palms by comparing the published genomic data of other palms. We sought to provide a significant and high quality genomic-level resource for further functional and evolutionary studies on this sole monocot mangrove species and palms in general.

## Results and Discussion

### *De novo* sequencing and assembly

**Fig 1:**
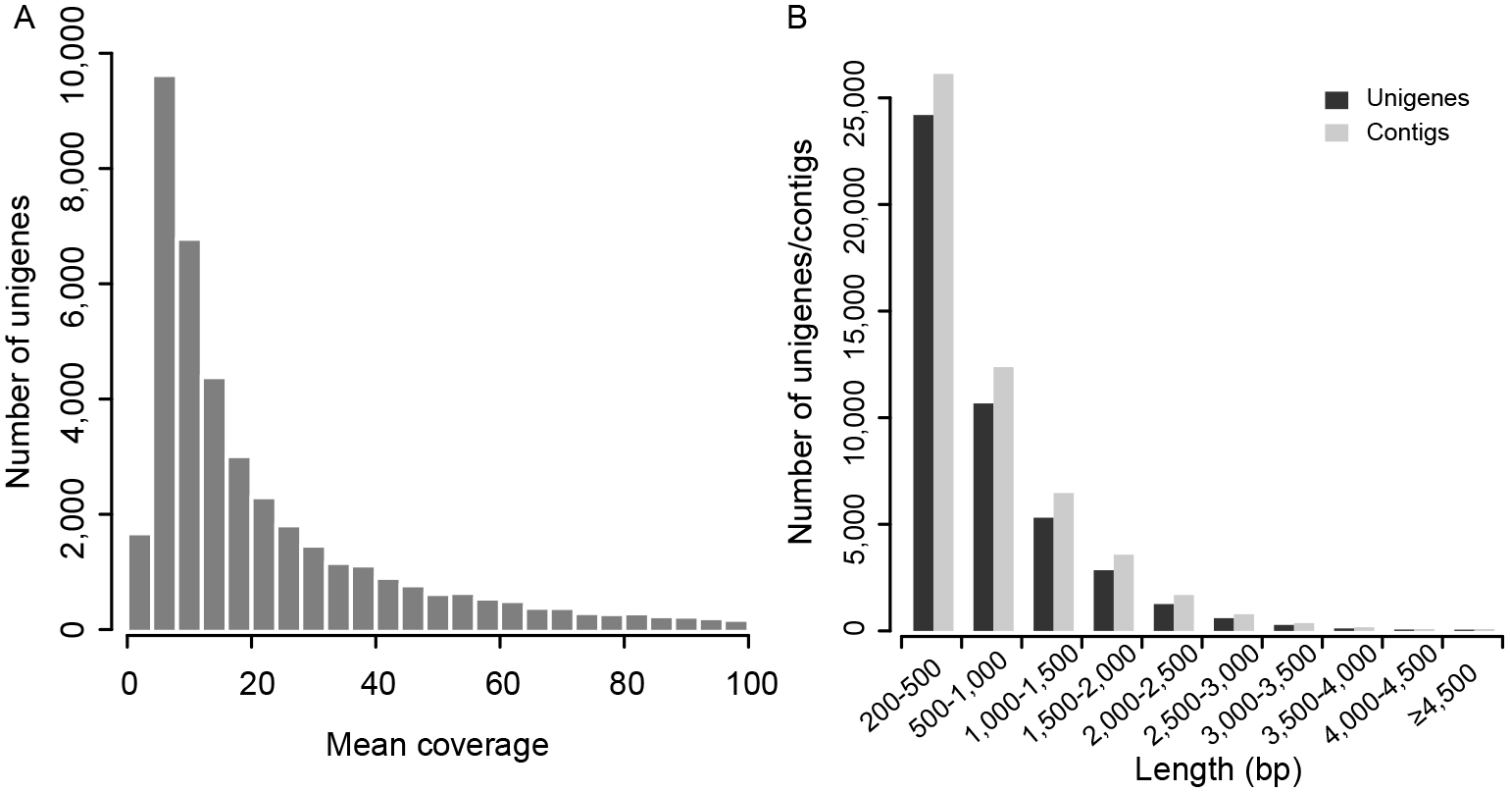
Distributions of the mean coverage of unigenes (a) and the length of unigenes and contigs (b).

To collect a large number of nucleotide sequences of coding regions, mRNAs of the fresh young leaves of one *N*. *fruticans* individual was isolated and sequenced via HiSeq 2000 platform. We obtained 24,607,427 raw paired-end reads and deposited them in the NCBI Short Read Archive (SRA) with the accession number SRR2016566. After filtering low quality reads, 19,918,800 clean reads were used and assembled into 51,702 contigs using Trinity [15]. Trinity includes three independent modules (Inchworm, Chrysalis and Butterfly) to assemble contigs step by step. It was widely used in the recent transcriptome analyses [10, 16, 17] because of its better performance in the quality of assembles compared to other computer programs [18]. We removed redundancy in these contigs, treated the remaining 45,368 contigs as unigenes and deposited them in NCBI GenBank under the accession number GDFT01000000. After mapping short reads to the unigenes, we calculated and plotted the mean coverage of each unigene (i.e., the average sequencing depth for all sites in a gene, Fig 1A). It showed that all unigenes were among the normal range compared to other studies [10, 16, 17]. The mean, median length and N50 value of the unigenes were 722 bp, 460 bp and 1,096 bp, respectively (Table 1). The N50 value indicates that half of the entire assembly is contained in sequences equal to or larger than this value. The high value of N50 indicates a high quality assembly. The N50 value of unigene was comparable to other studies (e.g. 956 bp of *Orchis italic* [16], 1,219 bp of *Cocos nucifera* [10] and 844 bp of *Ammopiptanthus mongolicus* [17]), indicating the good quality of the assembly. Total 21,220 unigenes were longer than 500 bp and were well assembled (Fig 1B). The GC content of *N*. *fruticans* was 47.7%, which was very close to that for the date palm *P*. *dactylifera* (47.6%) [19].

**Table 1:** Summary statistics of assembly and annotation for *Nypa fruticans*.

***De novo* assembly statistics**	
Total number of raw reads	24,607,427×2
Total number of clean reads	19,918,800×2
Total number of contigs	51,702
Unigenes (contigs after removing redundancy)	45,368
Mean length of unigenes (bp)	722
Median length of unigenes (bp)	460
N50 value of unigenes (bp)	1,096
Longest unigene (bp)	9,795
GC content	47.7%
**Annotation statistics of unigenes**	
NR-blast	32,260 (71.11%)
InterProScan	24,650 (54.33%)
Blast2GO	18,761 (41.35%)
KEGG pathway	2,956 (6.52%)
**SSR statistics**	
Di-nucleotide motifs	4,436 (55.74%)
Tri-nucleotide motifs	3,323 (41.76%)
Tetra-nucleotide motifs	174 (2.19%)
Penta-nucleotide motifs	10 (0.13%)
Hexa-nucleotide motifs	15 (0.19%)

The N50 value indicates that 50% of the entire assembly is contained in sequences equal to or larger than this value. NR: NCBI non-redundant protein database, InterProScan: a protein signature recognition tool, KEGG: Kyoto Encyclopedia of Genes and Genomes, SSR: simple sequence repeat.

### Annotation of the coding sequences

To obtain the functional annotation of each unigene, we firstly performed BLASTX against the NCBI non-redundancy database; 32,260 (71.11%) unigenes were matched (Table 1, S1 Table). Protein signature recognition was performed via InterProScan (http://www.ebi.ac.uk/interpro/), and a total of 24,650 (54.33%) unigenes had results. After merging the results of BLASTX with those of InterProScan, 18,761 (41.35%) unigenes were well-annotated via the analysis pipeline of Blast2GO (Table 1, S2 Table).

**Fig 2:**
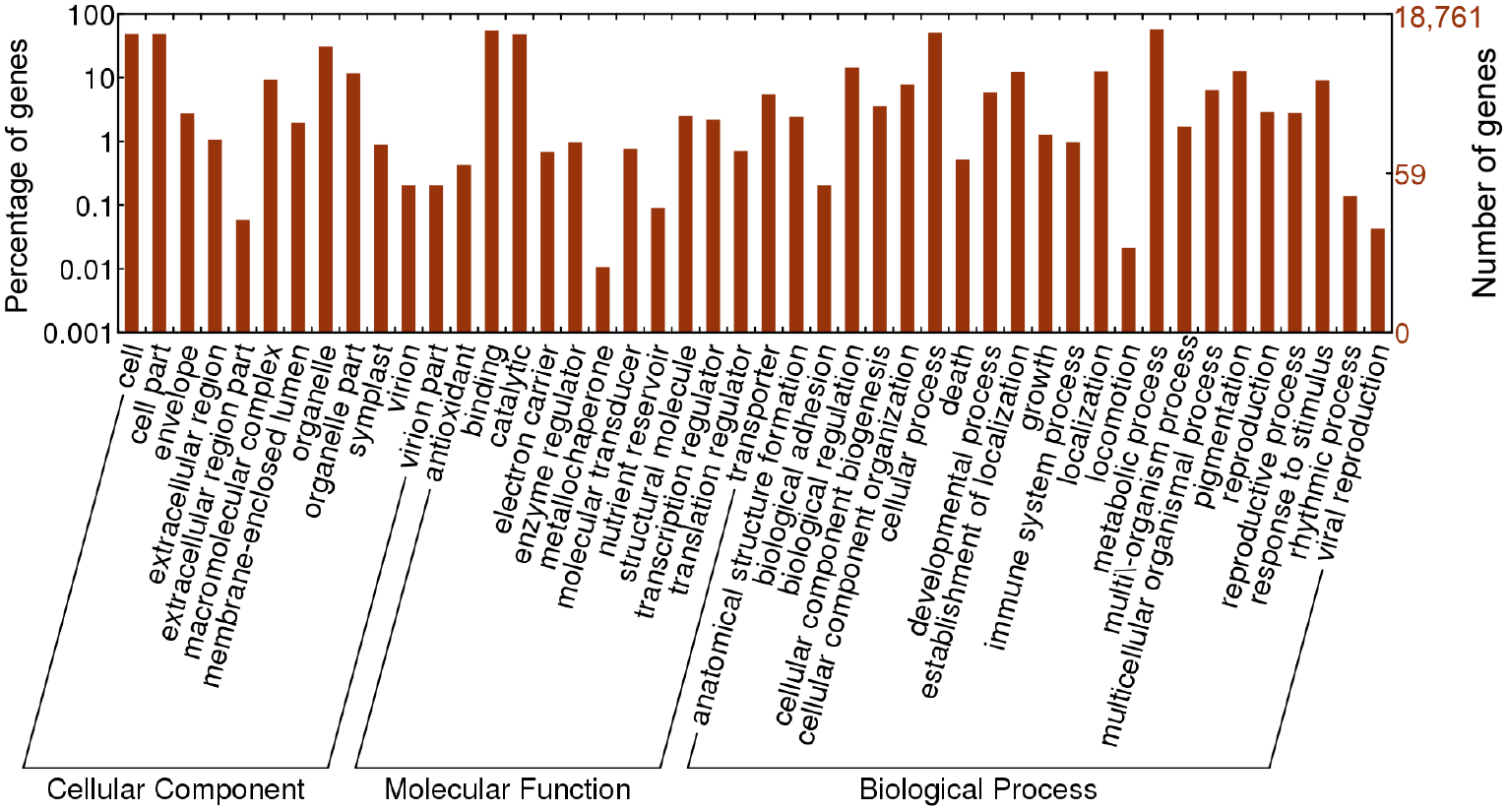
GO functional classification of *N*. *fruticans*. GO functional classification (level 2) of the annotated 18,761 unigenes.

Gene ontology (GO) is a major bioinformatics initiative to unify the representation of gene and gene product attributes. We retrieved GO annotations for 18,761 unigenes from the GO database and analyzed the enrichment of level-2 GO terms in three categories: cellular component, molecular function and biological process (Fig 2). For the cellular component category, ‘cell,’ ‘cell part’ and ‘organelle’ were the most abundant. For molecular function, ‘catalytic activity’ and ‘binding’ were overrepresented, and for the biological process, the two most highly represented categories were ‘metabolic process’ and ‘cellular process.’ In biological process, there were 1,198 unigenes annotated as “response to stress” (GO: 0006950). Among these, 131 unigenes were assigned to “response to osmotic stress” (GO: 0006970), 315 unigenes to “response to DNA damage stimulus” (GO: 0006974), and 178 unigenes to “response to oxidative stress” (GO: 0006979), all of which may be important for *N*. *fruticans*’ adaptation to an environment with high salinity, oxidation and UV light (S3 Table). Genes about “response to osmotic stress” were important for responding the increase or decrease of the concentration of solutes outside the organism or cell, which were helpful for *Nypa* to adapt to the high saline environment. Genes about “response to oxidative stress” would be important to reduce reactive oxygen species (ROS) and cell damage. When plants are subjected to stresses like high salinity, drought, high UV and flood, their balance between the productions of ROS and the quenching activity of antioxidants is upset, often resulting in oxidative damage. Genes about “response to DNA damage stimulus” would be related to dealing with the high UV conditions in the intertidal zone.

The pathway annotation was conducted via Kyoto Encyclopedia of Genes and Genomes (KEGG) analysis. The KEGG database contains data from a systematic analysis of inner-cell metabolic pathways and functions of gene products. Pathway-based analysis is helpful for understanding the biological functions and interactions of genes. In total, 2,956 unigenes were assigned to 134 pathways. The most enriched pathways were “Purine metabolism” (1,057 unigenes), “Thiamine metabolism” (865 unigenes) and “Biosynthesis of antibiotics” (450 unigenes; S4 Table and S1 Fig).

### Detecting positively selected genes

Understanding the adaptive evolution of species, especially non-model woody plants such as mangroves, has long been limited by the lack of genomic data. Despite their important ecological and evolutionary value, evidence for positive selection among mangroves and palms is scarce. Under positive selection, the frequency of advantageous allele increases because the individuals with this allele get a better phenotype to survival and reproduce more offspring than others. The positively selected genes are more likely fixed in the species. To detect the candidate positively selected genes, sequences of other species can be used to compare with the target species. Hence, we introduced the published genome sequences of *P*. *dactylifera*, *E*. *guineensis* and *O*. *sativa* to compare to the coding sequences of *N*. *fruticans* by the improved branch-site model. This model used the likelihood method to detect the candidate positively selected sites in the target species [20]. Among 2,136 single-copy orthologous genes, 15 putative positively selected genes were identified in Table 2. Four genes are of special interest. The orthologous gene of Nfr_c21523_g1_i2 in rice has a zinc finger domain, and its orthologous gene in *Arabidopsis thaliana* is involved in salinity stress [21]. The orthologous gene of Nfr_c19525_g1_i1 was also involved in response to salt stress [22]. These genes should be important for the adaptation of *N*. *fruticans* to a high-saline environment.

**Table 2:** Putative positively selected genes in *N*. *fruticans*.

Gene of *N*. *fruticans*	Orthologous gene in *O*. *sativa*	Orthologous gene in *A*. *thaliana*	Annotations
Nfr_c12132_g1_i1	LOC_Os06g36170.1	AT2G40570	initiator tRNA phosphoribosyl transferase family protein, putative, expressed
Nfr_CL1573Contig1	LOC_Os01g14610.2	AT3G12530	PSF2—putative GINS complex subunit, expressed
Nfr_CL1871Contig1	LOC_Os01g64990.1	--	GPI transamidase subunit PIG-U domain containing protein, expressed
Nfr_c17518_g1_i1	LOC_Os09g25950.1	AT4G28020	regulator protein, putative, expressed
Nfr_c32630_g1_i1	LOC_Os01g67100.1	AT1G65470	expressed protein
Nfr_c20436_g1_i2	LOC_Os01g46580.1	AT1G30825	actin-related protein 2/3 complex subunit 2, putative, expressed
Nfr_c22406_g1_i2	LOC_Os08g40590.2	AT4G25850, AT4G25860, AT5G57240	oxysterol-binding protein, putative, expressed
Nfr_c20352_g2_i1^a^	LOC_Os11g30560.1	AT5G50600, AT5G50700	dehydrogenase/reductase, putative, expressed
Nfr_c20446_g3_i1	LOC_Os02g03220.1	AT4G00800	protein-binding protein, putative, expressed
Nfr_c21523_g1_i2^a^	LOC_Os07g17400.1	AT5G58787, AT5G01520, AT1G24440	zinc finger, RING-type, putative, expressed
Nfr_c19525_g1_i1^a^	LOC_Os05g41100.1	AT3G24080	protein kri1, putative, expressed
Nfr_CL39Contig1	LOC_Os02g46750.1	AT5G16810	expressed protein
Nfr_c19922_g2_i1^a^	LOC_Os02g51480.1	AT4G39620	PPR repeat domain containing protein, putative, expressed
Nfr_c22757_g2_i2	LOC_Os06g06370.1	AT1G08030	expressed protein
Nfr_CL325Contig1	LOC_Os11g43610.1	AT4G02485	oxidoreductase, 2OG-Fe oxygenase family protein, putative, expressed

The positively selected genes were identified using the improved branch-site model. The Benjamini-Hochberg correction for multiple testing was used (FDR < 0.05).

^a^ These genes were functionally described in detail in the main text.

The other two genes may play important roles in the formation of vivipary in *N*. *fruticans*. The embryo of viviparous plants grows sufficiently to emerge visibly from within the seed tissue before dispersal. The formation of vivipary are related to the speed of embryo development, the regulation of seed dormancy and the mechanism of seed detaching from the parent. The *Arabidopsis* ortholog of Nfr_c19922_g2_i1 is essential for embryo development [23, 24], while the *Arabidopsis* ortholog of Nfr_c20352_g2_i1 (the hydroxysteroid dehydrogenase HSD1) was related to the lipid biosynthesis in seeds [25, 26]. The seed dormancy was terminated when HSD1 was overexpressed [26, 27]. These two genes about embryo development and seed dormancy may be related to the special feature vivipary. Since the two genes are related to other functions not only in embryo [27, 28], they also expressed in leaves with a normal expression level (the mean coverage of the two genes are 17.8 and 42.4, respectively).

Salt tolerance and vivipary germination are the most significant and fundamental adaptations for true mangroves to successfully occupy intertidal zones. Although *N*. *fruticans* and the compared palm species both originated in tropical and sub-tropical areas, they inhabit different ecological habitats—intertidal and terrestrial zones, respectively. Positive selection on these salinity stress and vivipary seeding–related genes may have contributed to woody plant adaptations to coastal environments.

### Identification of simple sequence repeats

**Fig 3:**
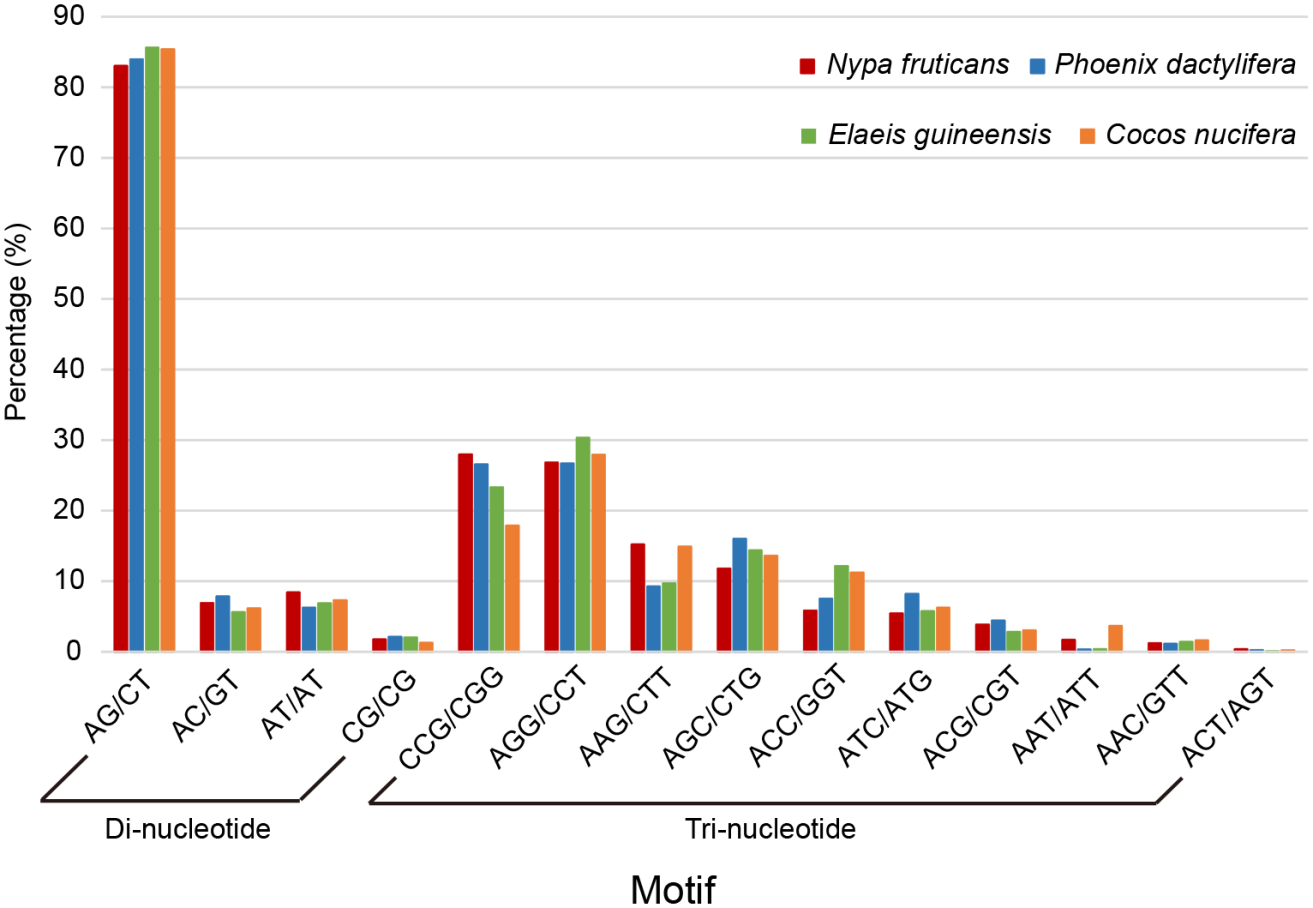
Frequency of different SSR motif types.

Simple sequence repeats (SSRs), also termed microsatellites, are nucleotide motif containing tandem repeat of two to five or six base pairs in DNA sequences. They are ubiquitous found in both protein coding and non-coding regions. SSRs are important molecular markers used in gene mapping, molecular breeding, genetic diversity, and discrimination. Based on the 45,382 unigenes, a total of 7,958 SSRs were identified in 6,541 unigenes (S5 Table). Approximately 14.4% transcriptomic sequences possess SSR loci (S5 Table). The mean SSR density was one per 4,122 bp. The most abundant type of repeat motif was di-nucleotide (55.74%), followed by tri-nucleotide (41.76%) and tetra-nucleotide (2.19%, Table 1). The percentages of penta-nucleotide and hexa-nucleotide were very low (0.13% and 0.19%, respectively). To further compare the motif frequency between different palms, we also detected the SSR motifs of three other palms (date palm *P*. *dactylifera*, oil palm *E*. *guineensis* and coconut *C*. *nucifera*) using their published genome and transcriptome data. In Fig 3, the motif frequencies of di-nucleotides showed a similar pattern among the four species: AG/CT was the most dominant (83.05%, 83.96%, 85.63% and 85.38% for *N*. *fruticans*, *P*. *dactylifera*, *E*. *guineensis* and *C*. *nucifera*, respectively) and CG/CG was the rarest. For tri-nucleotides, the most common motif for *N*. *fruticans* and *P*. *dactylifera* was CCG/CGG (27.96% and 26.55%), whereas that for *E*. *guineensis* and *C*. *nucifera* was AGG/CCT (30.28% and 27.90%).

To validate the predicted SSRs, total 36 pairs of PCR primers for the randomly picked SSRs were designed and used for PCR. Twenty-five of them successfully got the PCR products. We sequenced these 25 PCR products using Sanger sequencing. All sequencing results were the same as the genes assembled by Trinity. The predicted SSRs were 100% validated (S6 Table). The total 25 sequenced gene segments are all homozygote, suggesting the low heterozygosity of *N*. *fruticans*.

### Phylogenetic tree and divergence time

**Fig 4:**
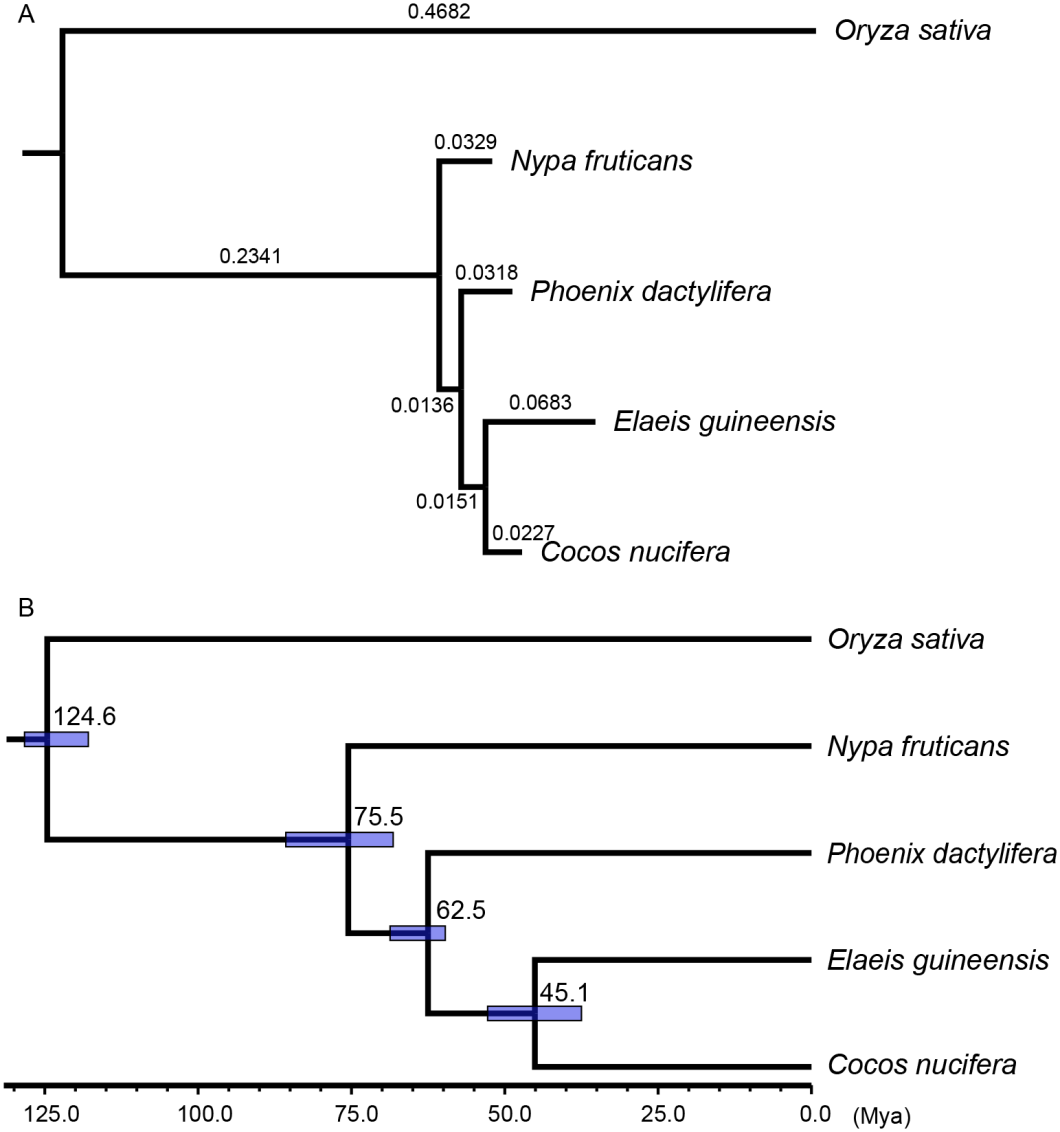
Phylogenetic analysis and divergence time estimation. (A) The phylogenetic tree of the five monocots. The results are 100% supported by the 1,000 bootstraps analysis. (B) Estimation of divergence time. Blue bars indicate 95% confidence intervals.

*Nypa* represents an independent line of specialization within the palm family and has no discernable close relatives due to its numerous distinctive characteristics [1]. *Nypa* has even been suggested as a distinct family (Nypaceae) [1]. To perform a phylogenetic analysis, we collected and downloaded the genomes and transcriptomes of four other species: the three sequenced palms (*P*. *dactylifera*, *E*. *guineensis*, *C*, *nucifera*) and *Oryza sativa* as an out-group species. A total of 22,168 ortholog groups were generated using OrthoMCL software [29], and 1,347 single-copy orthologs were retrieved to reconstruct a phylogenetic tree and calculate the divergence time. The phylogenetic tree was drawn with the best-fit model (Fig 4A). The phylogenetic tree was 100% supported by 1,000 bootstraps analysis. It showed that *N*. *fruticans* was the species divergent from the ancestor of other three palms, which was consist with previous studies. The branch lengths of palms were much shorter than that of *O*. *sativa*, suggesting the mutation rate became smaller in the lineage of palms. The divergence time between *N*. *fruticans* and other palms was estimated at approximately 75.5 Mya (million years ago) (Fig 4B).

The paleobiogeography of *Nypa* has long been intensively investigated because it is one of the oldest palms and one of the first monocots in the fossil plant record [2, 5]. Our estimation of its divergence time from other palm relatives, as well as its first appearance in the form of pollen in the Campanian (Late Cretaceous, 72.1–83.6 Mya [5]), indicate the fact that this genus firstly appeared at least in Late Cretaceous. *Nypa*, as well as *Acrostichum*, *Pelliceria* and now extinct species *Brevitricolpites variabilis*, are the first members found to inhabit the brackish water environment [2]. The earliest unequivocal fossil palm materials probably dated from the Early to Mid-Late Cretaceous, and the expanding and retracting distribution of fossils in higher latitudes have been important indicators of global changes [6]. The large number of fossils of *N*. *fruticans* showed maximum expansion during the Early Eocene, perhaps corresponding with the climate optimum. However, the cooling temperature and seasonal climate reduced its cosmopolitan range. This species made its last appearance in the New World in the Late Eocene and it is currently restricted to the Old World [2].

### Whole-genome duplication events

Whole-genome duplication events could provide functional and evolutionary materials for speciation and adaptation. A previous study found two genome-wide duplication events of the date palm *P*. *dactylifera* [8]. Whether *N*. *fruticans* share these two WGD events is an interesting question. The distribution of synonymous substitution rate (number of synonymous nucleotide substitution per synonymous sites, Ks) of paralogs could be used for identification of WGD event. The larger Ks, the longer divergence time between paralogs. For a species that experienced a WGD event, most genomic sequences were doubled at the same time. The mutations were then accumulated between the paralogs introduced by WGD. The peak of Ks distribution would be a good indicator of WGD event, since it state a large number of genes duplicated in a narrow time range.

**Fig 5:**
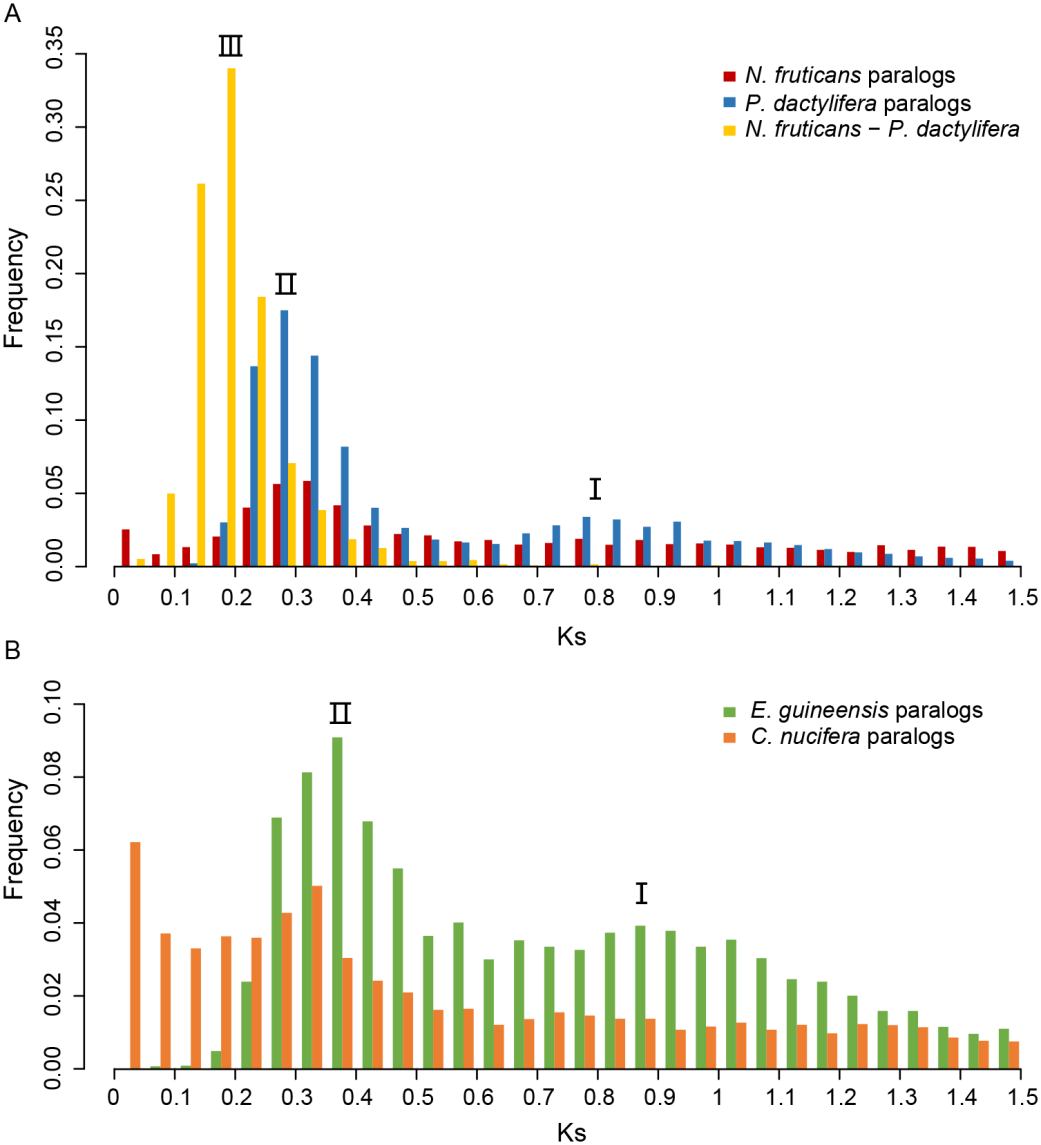
Whole-genome duplication events of four palms. (A) The distribution of synonymous substitution rate (Ks) of the paralogs is displayed in red and blue for *N*. *fruticans* and *P*. *dactylifera*, respectively. Peaks I and II indicated two whole-genome duplication events in both species. The yellow bars showed the distribution of Ks of the orthologs between the two species. Peak III indicated that the divergence time of the two palms was close to the recent duplication events (peak II). (B) The distribution of Ks of paralogs of *E*. *guineensis* and *C*. *nucifera*. The same two whole-genome duplication events can be found for the two species.

In Fig 5A, the two peaks (I and II) of the Ks distribution of *P*. *dactylifera* (blue bars) showed two WGD events, as is consistent with the descriptions in Al-Mssallem *et al*. [8]. The red bars indicated Ks values of the paralogs of *N*. *fruticans*, suggesting that the two WGD events occurred near the same time of *P*. *dactylifera* despite the fact that peak I of *N*. *fruticans* was weaker than that of *P*. *dactylifera*. Peak I shows that the generally accepted duplication event of flowering plants happened approximately 150 Mya [30], whereas peak II on the left suggested a recent WGD event. Peak III of the Ks of orthologs between the two palms (yellow bars) is to the left of the peak II, suggesting that the two palms shared the same two WGD events and diverged shortly after the recent duplication event. To investigate the WGDs in other palms, we calculated and showed the Ks distributions of *E*. *guineensis* and *C*. *nucifera* (Fig 5B). *E*. *guineensis* (green bars) showed two WGD events as *P*. *dactylifera*; the two peaks were slightly right-shifted compared with those of *P*. *dactylifera*. This may have been due to the relatively high substitution rate and could be consistent with the longer branch of *E*. *guineensis* in Fig 4A. The Ks distribution of *C*. *nucifera* produced peak II, whereas peak I was too weak to detect. The transcriptome redundancy of *C*. *nucifera* may contribute to the leftmost peak being near zero. In summary, we detected two WGD events—an ancient one (approximately 150 Mya) and a recent one (prior to the divergence of palms, which likely occurred in the common ancestor of all palms) based on the available resources.

If the ancient WGD event was dated to approximately 150 Mya and the synonymous substitution rate has remained constant, the divergence time of the two palms should be approximately 40 Mya, which differs from our divergence estimation and fossil records. Hence, the mutation rate of palms had been decreasing during their evolution, which may be associated with a shift to the tree/shrub habit [31]. The short branch lengths of the four palms also support this conclusion (Fig 4A).

## Conclusion

*N*. *fruticans* is the only monocot species that has adapted to intertidal zones. In this study, we provide a well-annotated transcriptome of *N*. *fruticans* with 45,368 unigenes, of which 71.11% had BLASTX hits and 41.35% were annotated in the Blast2GO pipeline. Several positively selected genes related to salt tolerance and vivipary development were detected. These genes may be important for its adaptation. We also detected two WGD events that are shared among four palms, and the divergence of these palms occurred shortly after the recent WGD event. Because *N*. *fruticans* is the basal species in the family Arecaceae (exclude subfamily Calamoideae), these two WGD events likely occurred in the common ancestor of all palms, which provided a large amount of raw material for the following more than 2,000 speciation events in Arecaceae. This study provides a high quality resource for further functional and evolutionary studies of *N*. *fruticans* and palms in general.

## Materials and Methods

### Ethics statement

All of the necessary permits for field studies were obtained. The Hainan Dongzhaigang National Nature Reserve Authority provided permission to collect the samples for our research.

### Plant materials and RNA sequencing

*N*. *fruticans* used in this study was collected from Dongzhai Harbor, Haikou, Hainan, China (19°57'12" N; 110°33'59" E). Since the main object of the study is not to compare the expression level of genes but to generate a large number of nucleotide sequences of *N*. *fruticans* to do the following phylogenetic and other analysis, only one transcriptome was sequenced. Total RNA was isolated from the fresh young leaves of one individual using the modified CTAB method and precipitated with 5 M LiCl at -20°C overnight. The resulting RNA pellets were suspended in 70 μL DEPC-treated water. The quantified total RNA (concentration > 500 ng/μL; rRNA ratio  =  1.2) was delivered to the Beijing Genome Institute (Shenzhen, China) for further treatment. The mRNAs were extracted from the total RNAs using Oligotex^TM^-dT30 (TaKaRa, Dalian, China) and then fragmented ultrasonically and converted to double-stranded cDNAs using random primers. A nucleotide “A” was added at the 3’-end of cDNAs, and then adapters were ligated to both ends. The purifying cDNAs were sequenced via HiSeq 2000 (Illumina Inc., San Diego, CA, USA) with a read length of 90 bp and insertion size of 200 bp.

### *De novo* assembly and annotation

The raw reads were filtered using SolexaQA [32] with the following steps: the raw reads were trimmed via DynamicTrim (quality threshold = 20) and then filtered using LengthSort (length cutoff = 50 bp). The clean reads were *de novo* assembled into contigs using Trinity [15] with the default parameters except for “min_kmer_cov = 2”. We removed the redundancy of the assembled contigs using TGICL [33] and CD-HIT [34]. The clean reads were then mapped to the retained contigs using bowtie2 [35], and their mean coverage was calculated. Contigs with a mean coverage of less than two were filtered out. The remaining contigs were treated as unigenes.

To obtain the functional annotation of *N*. *fruticans*, the unigenes were firstly searched against the NCBI non-redundancy (NR) protein database using BLASTX with an e-value threshold of 10^−6^; the results were then evaluated using Blast2GO (v.3.0.0 PRO) [36] for the downstream analyses. The distribution of level-2 Gene Ontology (GO) terms of the annotated unigenes was plotted using WEGO for biological processes, molecular functions and cellular components categories [37].

### SSRs identification and validation

We used MISA (MIcroSAtellite identification tool, http://pgrc.ipk-gatersleben.de/misa/) to identify the simple sequence repeat (SSR). The SSRs were considered to contain motifs with two to six nucleotides and a minimum of five contiguous repeat units.

To validate the predicted SSRs, we used Primer3 software [38] to design primers around the SSRs. The key parameters set for primer design were as follows: primer length 18–24 bp, PCR product size 100–280 bp, optimum annealing temperature 55–60°C and GC content 40%-65% with 50% as the optimum. We random picked 36 pairs of them to do the PCR experiment. PCR were set up in a 30μl reaction mixture in PCR tubes. Each reaction mixture contained 0.4 μM each forward and reverse primer, 0.4 mM each dNTP, 1.5 U LA Taq DNA polymerase and 3μl 10x LA Taq buffer (TaKaRa, Japan) and 50–100 ng of genomic DNA as a template. PCR amplification was carried out using a Dyad Disciple thermal Cycler (Bio-Rad, Beijing, USA) with the following cycling conditions: pre-denaturation at 94°C for 5 min followed by 33 cycles of 94°C for 30 sec, 57°C for 45 sec and 72°C for 1 min 30 sec, and finally, 10 min at 72°C for extension. Twenty-five of them successfully got the PCR products and were sequenced by Sanger sequencing.

### Identification of candidate positively selected genes

We combined the genome sequences of *P*. *dactylifera*, *E*. *guineensis* and *O*. *sativa* to cluster gene families using the OrthoMCL software [29] and obtained 2,136 single-copy genes. We then detected positively selected genes using CODEML in the PAML 4.8 package [39]. Setting *N*. *fruticans* as the foreground branch, we used the improved branch-site model to calculate the likelihood of the null model (no sites in the foreground were positively selected) and alternative model (existing sites in the foreground were positively selected) to obtain the likelihood ratio. To remove false discoveries, the Benjamini-Hochberg correction for multiple testing was used (FDR < 0.05). Positively selected genes were also annotated based on the annotation of the orthologs of *O*. *sativa* (MSU Rice Genome Annotation Project). The orthologs of *A*. *thaliana* were adopted from the orthologous groups of *O*. *sativa* in MSU Rice Genome Annotation Project (http://rice.plantbiology.msu.edu) and The Arabidopsis Information Resource (https://www.arabidopsis.org). For the genes with no record in the databases, we used Blastx with an e-value of 10^−6^ to find the orthologs.

### Identification of ortholog groups and divergence time estimation

The OrthoMCL software was used to cluster the ortholog groups of five monocots, including four palms (*N*. *fruticans*, *P*. *dactylifera*, *E*. *guineensis*, *C*. *nucifera*) and the out-group species *Oryza sativa* [40]. We firstly extracted the coding region sequences (CDS) of these unigenes based on the BLASTX results and then translated CDS to proteins using a Bioperl script. All-versus-all BLASTP was conducted for all protein sequences with a cutoff of 10^−10^, and hits with identities of less than 40% were removed. The results were fed to OrthoMCL software to obtain ortholog groups with default settings. Protein sequences of each single-copy ortholog from the OrthoMCL results were retrieved and aligned using MUSCLE [41]. The protein alignments were then converted to nucleotide alignments using pal2nal [42]. Any alignment shorter than 150 bp was removed from the analysis.

To reconstruct the phylogenetic tree, we first performed a model test using jmodeltest2 [43]. The best-fit model was GTR+G. The phylogenetic tree was then reconstructed using PhyML3.0 [44] with the best-fit model and 1,000 bootstraps. We estimated the divergence time of each node using MCMCTREE of the PAML 4.8 package [39]. The time constraints were set as follows: 1) the divergence time between *N*. *fruticans* and *O*. *sativa* was set to 117–128 Mya because of the stem node age of Araceae and Poales [45]; 2) the divergence time of *P*. *dactylifera* and *E*. *guineensis* was set to 60–70 Mya [9].

### Detection of whole-genome duplication events

The protein sequences of *N*. *fruticans* and *C*. *nucifera* were used to conduct BLASTP against itself. Then, the OrthoMCL software was used to obtain the paralogs of *N*. *fruticans*. For the published genomes of *P*. *dactylifera* and *E*. *guineensis*, we used the synteny genes as paralogs. The methods of sequence alignment and filtering criteria are the same as those in the previous section. The synonymous substitution rate of each paralog for each species were calculated using the KaKs-Calculator [46] with the NG (Nei-Gojobori) model.

## Supporting Information

S1 Fig The summary of KEGG pathway. (TIF)

S1 Table Summary of NR top blast results. (XLSX)

S2 Table Gene ontology and annotation of unigenes. (XLSX)

S3 Table Unigenes of *N*. *fruticans* with GO terms about response to stress. (XLSX)

S4 Table Summary of KEGG pathways. (XLSX)

S5 Table Information of SSRs identified in *N*. *fruticans*. (XLSX)

S6 Table SSR primers for SSRs validation. (XLSX)

## References

[1] Tomlinson P. The botany of mangroves: Cambridge University Press, Cambridge; 1986pp. 147–151, 295–303.

[2] Gee CT. The mangrove palm Nypa in the geologic past of the New World. Wetlands Ecol Manage. 2001; 9: 181–203.

[3] Asmussen CB, Dransfield J, Deickmann V, Barfod AS, PINTAUD JC, Baker WJ. A new subfamily classification of the palm family (Arecaceae): evidence from plastid DNA phylogeny. Bot J Linn Soc. 2006; 151: 15–38.

[4] Sunderland T, Morakinyo T. Nypa fruticans, a weed in West Africa. Pamls. 2002; 44: 154–155.

[5] Schrank E. Palaeozoic and Mesozoic palynomorphs from northeast Africa (Egypt and Sudan) with special reference to Late Cretaceous pollen and dinoflagellates. Berliner Geowissenschaftliche Abhandlungen A. 1987; 75: 249–310.

[6] Harley MM. A summary of fossil records for Arecaceae. Bot J Linn Soc. 2006; 151: 39–67.

[7] Jian S, Ban J, Ren H, Yan H. Low genetic variation detected within the widespread mangrove species Nypa fruticans (Palmae) from Southeast Asia. Aquat Bot. 2010; 92: 23–27.

[8] Al-Mssallem IS, Hu S, Zhang X, Lin Q, Liu W, Tan J, et al Genome sequence of the date palm Phoenix dactylifera L. Nat Commun. 2013; 4. 10.1038/ncomms3274 PMC3741641 23917264

[9] Singh R, Ong-Abdullah M, Low E-TL, Manaf MAA, Rosli R, Nookiah R, et al Oil palm genome sequence reveals divergence of interfertile species in Old and New worlds. Nature. 2013; 500: 335–339. 10.1038/nature12309 23883927 PMC3929164

[10] Fan H, Xiao Y, Yang Y, Xia W, Mason AS, Xia Z, et al RNA-Seq analysis of Cocos nucifera: transcriptome sequencing and de novo assembly for subsequent functional genomics approaches. PLoS One. 2013; 8: e59997 10.1371/journal.pone.0059997 23555859 PMC3612046

[11] Wood TE, Takebayashi N, Barker MS, Mayrose I, Greenspoon PB, Rieseberg LH. The frequency of polyploid speciation in vascular plants. Proc Natl Acad Sci U S A. 2009; 106: 13875–13879. 10.1073/pnas.0811575106 19667210 PMC2728988

[12] Vanneste K, Baele G, Maere S, Van de Peer Y. Analysis of 41 plant genomes supports a wave of successful genome duplications in association with the Cretaceous-Paleogene boundary. Genome Res. 2014; 24: 1334–1347. 10.1101/gr.168997.113 24835588 PMC4120086

[13] Shi T, Huang H, Barker MS. Ancient genome duplications during the evolution of kiwifruit (Actinidia) and related Ericales. Ann Bot. 2010; 106: 497–504. 10.1093/aob/mcq129 20576738 PMC2924827

[14] McKain MR, Wickett N, Zhang Y, Ayyampalayam S, McCombie WR, Chase MW, et al Phylogenomic analysis of transcriptome data elucidates co-occurrence of a paleopolyploid event and the origin of bimodal karyotypes in Agavoideae (Asparagaceae). Am J Bot. 2012; 99: 397–406. 10.3732/ajb.1100537 . 22301890

[15] Grabherr MG, Haas BJ, Yassour M, Levin JZ, Thompson DA, Amit I, et al Full-length transcriptome assembly from RNA-Seq data without a reference genome. Nat Biotechnol. 2011; 29: 644–652. 10.1038/nbt.1883 21572440 PMC3571712

[16] De Paolo S, Salvemini M, Gaudio L, Aceto S. De novo transcriptome assembly from inflorescence of Orchis italica: analysis of coding and non-coding transcripts. PLoS One. 2014; 9: e102155 10.1371/journal.pone.0102155 25025767 PMC4099010

[17] Gao F, Wang J, Wei S, Li Z, Wang N, Li H, et al Transcriptomic Analysis of Drought Stress Responses in Ammopiptanthus mongolicus Leaves Using the RNA-Seq Technique. PLos One. 2015; 10: e0124382 10.1371/journal.pone.0124382 25923822 PMC4414462

[18] Zhao Q-Y, Wang Y, Kong Y-M, Luo D, Li X, Hao P. Optimizing de novo transcriptome assembly from short-read RNA-Seq data: a comparative study. BMC Bioinformatics. 2011; 12: S2. 10.1186/1471-2105-12-S14-S2 PMC3287467 22373417

[19] Al-Dous EK, George B, Al-Mahmoud ME, Al-Jaber MY, Wang H, Salameh YM, et al De novo genome sequencing and comparative genomics of date palm (Phoenix dactylifera). Nat Biotechnol. 2011; 29: 521–527. 10.1038/nbt.1860 21623354

[20] Zhang J, Nielsen R, Yang Z. Evaluation of an improved branch-site likelihood method for detecting positive selection at the molecular level. Mol Biol Evol. 2005; 22: 2472–2479. 16107592 10.1093/molbev/msi237

[21] Gong Q, Li P, Ma S, Indu Rupassara S, Bohnert HJ. Salinity stress adaptation competence in the extremophile Thellungiella halophila in comparison with its relative Arabidopsis thaliana. The Plant Journal. 2005; 44: 826–839. 16297073 10.1111/j.1365-313X.2005.02587.x

[22] Lamesch P, Berardini TZ, Li D, Swarbreck D, Wilks C, Sasidharan R, et al The Arabidopsis Information Resource (TAIR): improved gene annotation and new tools. Nucleic Acids Res. 2012; 40: D1202–D1210. 10.1093/nar/gkr1090 22140109 PMC3245047

[23] Beick S, Schmitz-Linneweber C, Williams-Carrier R, Jensen B, Barkan A. The pentatricopeptide repeat protein PPR5 stabilizes a specific tRNA precursor in maize chloroplasts. Mol Cell Biol. 2008; 28: 5337–5347. 10.1128/MCB.00563-08 18591259 PMC2519714

[24] Cushing DA, Forsthoefel NR, Gestaut DR, Vernon DM. Arabidopsis emb175 and other ppr knockout mutants reveal essential roles for pentatricopeptide repeat (PPR) proteins in plant embryogenesis. Planta. 2005; 221: 424–436. 15647901 10.1007/s00425-004-1452-x

[25] O'Neill C, Morgan C, Hattori C, Brennan M, Rosas U, Tschoep H, et al Towards the genetic architecture of seed lipid biosynthesis and accumulation in Arabidopsis thaliana. Heredity. 2012; 108: 115–123. 10.1038/hdy.2011.54 21731053 PMC3262871

[26] Baud S, Dichow NR, Kelemen Z, d’Andréa S, To A, Berger N, et al Regulation of HSD1 in seeds of Arabidopsis thaliana. Plant and cell physiology. 2009; 50: 1463–1478. 10.1093/pcp/pcp092 19542545

[27] Li F, Asami T, Wu X, Tsang EW, Cutler AJ. A putative hydroxysteroid dehydrogenase involved in regulating plant growth and development. Plant Physiol. 2007; 145: 87–97. 17616511 10.1104/pp.107.100560 PMC1976581

[28] Schmid M, Davison TS, Henz SR, Pape UJ, Demar M, Vingron M, et al A gene expression map of Arabidopsis thaliana development. Nat Genet. 2005; 37: 501–506. 15806101 10.1038/ng1543

[29] Li L, Stoeckert CJ Jr., Roos DS. OrthoMCL: identification of ortholog groups for eukaryotic genomes. Genome Res. 2003; 13: 2178–2189. 10.1101/gr.1224503 12952885 PMC403725

[30] Van de Peer Y, Fawcett JA, Proost S, Sterck L, Vandepoele K. The flowering world: a tale of duplications. Trends Plant Sci. 2009; 14: 680–688. 10.1016/j.tplants.2009.09.001 19818673

[31] Smith SA, Donoghue MJ. Rates of molecular evolution are linked to life history in flowering plants. science. 2008; 322: 86–89. 10.1126/science.1163197 18832643

[32] Cox MP, Peterson DA, Biggs PJ. SolexaQA: At-a-glance quality assessment of Illumina second-generation sequencing data. BMC Bioinformatics. 2010; 11: 485 10.1186/1471-2105-11-485 . 20875133 PMC2956736

[33] Pertea G, Huang X, Liang F, Antonescu V, Sultana R, Karamycheva S, et al TIGR Gene Indices clustering tools (TGICL): a software system for fast clustering of large EST datasets. Bioinformatics. 2003; 19: 651–652. 12651724 10.1093/bioinformatics/btg034

[34] Fu L, Niu B, Zhu Z, Wu S, Li W. CD-HIT: accelerated for clustering the next-generation sequencing data. Bioinformatics. 2012; 28: 3150–3152. 10.1093/bioinformatics/bts565 23060610 PMC3516142

[35] Langmead B, Salzberg SL. Fast gapped-read alignment with Bowtie 2. Nat Meth. 2012; 9: 357–359. 10.1038/nmeth.1923 PMC3322381 22388286

[36] Conesa A, Gotz S, Garcia-Gomez JM, Terol J, Talon M, Robles M. Blast2GO: a universal tool for annotation, visualization and analysis in functional genomics research. Bioinformatics. 2005; 21: 3674–3676. 10.1093/bioinformatics/bti610 16081474

[37] Ye J, Fang L, Zheng H, Zhang Y, Chen J, Zhang Z, et al WEGO: a web tool for plotting GO annotations. Nucleic Acids Res. 2006; 34: W293–W297. 10.1093/nar/gkl031 16845012 PMC1538768

[38] Koressaar T, Remm M. Enhancements and modifications of primer design program Primer3. Bioinformatics. 2007; 23: 1289–1291. 17379693 10.1093/bioinformatics/btm091

[39] Yang Z. PAML 4: phylogenetic analysis by maximum likelihood. Mol Biol Evol. 2007; 24: 1586–1591. 10.1093/molbev/msm088 . 17483113

[40] Ouyang S, Zhu W, Hamilton J, Lin H, Campbell M, Childs K, et al The TIGR rice genome annotation resource: improvements and new features. Nucleic Acids Res. 2007; 35: D883–D887. 10.1093/nar/gkl976 17145706 PMC1751532

[41] Edgar RC. MUSCLE: multiple sequence alignment with high accuracy and high throughput. Nucleic Acids Res. 2004; 32: 1792–1797. 10.1093/nar/gkh340 15034147 PMC390337

[42] Suyama M, Torrents D, Bork P. PAL2NAL: robust conversion of protein sequence alignments into the corresponding codon alignments. Nucleic Acids Res. 2006; 34: W609–W612. 10.1093/nar/gkl315 16845082 PMC1538804

[43] Darriba D, Taboada GL, Doallo R, Posada D. jModelTest 2: more models, new heuristics and parallel computing. Nat Methods. 2012; 9: 772. 10.1038/nmeth.2109 PMC4594756 22847109

[44] Guindon S, Dufayard JF, Lefort V, Anisimova M, Hordijk W, Gascuel O. New algorithms and methods to estimate maximum-likelihood phylogenies: assessing the performance of PhyML 3.0. Syst Biol. 2010; 59: 307–321. 10.1093/sysbio/syq010 . 20525638

[45] Janssen T, Bremer K. The age of major monocot groups inferred from 800+ rbcL sequences. Bot J Linn Soc. 2004; 146: 385–398.

[46] Zhang Z, Li J, Zhao X-Q, Wang J, Wong GK-S, Yu J. KaKs_Calculator: Calculating Ka and Ks Through Model Selection and Model Averaging. Genomics, Proteomics & Bioinformatics. 2006; 4: 259–263. 10.1016/s1672-0229(07)60007-2 PMC5054075 17531802

