# Supplementary figures and images for: De Novo Assembly of Coding Sequences of the Mangrove Palm (Nypa fruticans) Using RNA-Seq and Discovery of Whole-Genome Duplications in the Ancestor of Palms
Source: PLoS One. 2015 Dec 18;10(12):e0145385. doi: 10.1371/journal.pone.0145385 (PMC4684314; doi:10.1371/journal.pone.0145385)

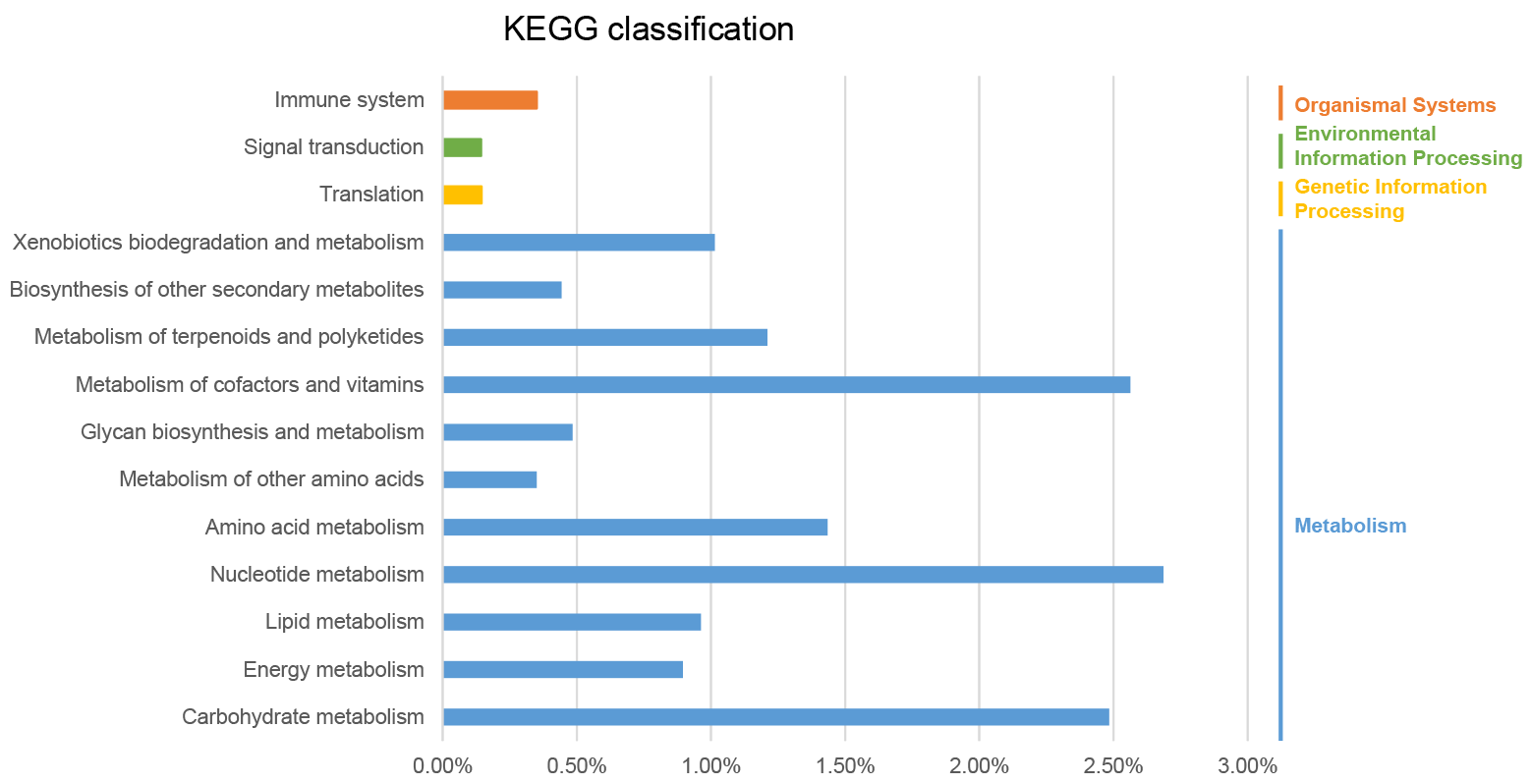

Supplement: S1 Fig — (TIF) [file pone.0145385.s001.tif]
